# Remarks on the Sirocco, or South-East Wind of the Mediterranean—Climate of Sicily—Consumption in That Island

**Published:** 1815-11

**Authors:** John C. Yeatman

**Affiliations:** Member of the Royal College of Surgeons, London.


THE LONDON

### Medical and Physical Journal.

5 OF VOL. XXXIV.] NOVEMBER, 1815. [NO. 201.

"For many fortunate discoveries in medicine, and for the detection of numerous errors, the world is indebted to the rapid circulation of Monthly Journals; and there never existed any work to which the faculty in EUROPE and AMERICA were under deeper obligations than to the Medical and Physical Journal of London, now forming a long, but an invaluable, series."—RUSH.

*For the London Medical and Physical Journal.*

### *Remarks on the Sirocco, or South-East Wind of the Mediterranean—Climate of Sicily—Consumption in that Island.* By JOHN C. YEATMAN, Esq. Member of the Royal College of Surgeons, London.

FINDING, by the late Numbers of your Journal, that Drs. Sutton and Gladstone are in error concerning the Sirocco wind, and supposing that its properties are unknown to your readers in general, I have been induced to give the following practical account of it.

The Sirocco is a hot, humid,* hazy blast, felt at intervals during the months of June, July, August, and September, in Sicily and other parts of the Mediterranean, and continuing usually three, sometimes seven, and even ten days together. I have frequently observed this singular wind from my hospital at the Convent of Capuchins, (situated on the side of the primitive hills over and above the city and straits of Messina,) diffusing itself in the atmosphere and enveloping this part of the island in mist like the morning vapour of a vast lake. In a moment from enjoying the invigorating influence of a tramontane, or north wind, we are exposed to an atmosphere which causes relaxation of muscular fibre, and a more or less depression of animal spirits,† and occasions a moisture

* "The south-east wind is the celebrated Sirocco, whose qualities, however, have been greatly exaggerated. It seems to derive its peculiarities from its superior *heat and dampness*."—*Irvine on the Diseases in Sicily*, page 6.

† Brydone, in his Tour through Sicily and Malta, gives the following exaggerated account of the effect of the Sirocco on the animal spirits. Mr. B. though sometimes an intelligent writer, oftener succeeds in pleasing the taste of his readers with the flowing and a moisture upon the skin which, mingling with the perspiration, gives to the body a cold and clammy sensation. The robust native* of more northern Europe scarcely, however, feels its depressing influence, whilst the delicate Italian and the debilitated invalid complain of the languor it occasions. I did not feel this effect upon my spirits till the second year of my residence in Sicily, but was informed by the inhabitants that I should complain more of ennui from the Sirocco after a longer stay in the kingdom. This I found, from some old

---

and lively skill of epistolary composition, than in satisfying their judgment with the accuracy of his statements. He says, "It is infinitely more relaxing, and gives the vapours in a much stronger degree than the worst of our rainy Novembers. It has now blown for these seven days without intermission; and has, indeed, blown away all our gaiety and spirits, and, if it continues much longer, I do not know what may be the consequence. It gives a degree of lassitude, both to the body and mind, that renders them absolutely incapable of performing their usual functions. It is not very surprising that it should produce these effects in a phlegmatic English constitution; but we have just now an instance, that all the mercury of France must sink under the load of this horrid leaden atmosphere. A smart Parisian Marquiss came here about ten days ago: he was so full of animal spirits, that the people thought him mad. He never remained a moment in the same place; but, at their grave conversations, he used to skip about from room to room with such amazing elasticity that the Italians swore he had got springs in his shoes. I met him this morning with the step of a philosopher, a smelling bottle in his hand, and all his vivacity extinguished. I asked, what was the matter? 'Ah! mon ami, (said he,) je m'ennui a la mort;—moi, qui n'ai jamais seu l'ennui. Mais cet execrable vent m'accable et deux jours de plus et je me pend.'

"The natives themselves do not suffer less than strangers, as all nature seems to languish during this abominable wind. A Neapolitan lover avoids his mistress with the utmost care in the time of the Sirocco, and the indolence it inspires is almost sufficient to extinguish every passion; all works of genius are laid aside during its continuance; and, when any thing very flat or insipid is produced, the strongest phrase of disapprobation they can bestow is 'Era scritto in tempo del Sirocco,'—that it was writ in the time of the Sirocco."

* "The Sirocco winds in Sicily are certainly, and particularly on the northern coast, a very great grievance; and, although they have been found to occasion no unpleasant sensations to *certain foreigners of a perfect temperament*, yet, to the common run of mankind, a temperature of 90, 100, or even 106 degrees, is not much to their taste."—*A View of the present State of Sicily from Abbate Balsamo, by Vaughan.*

English

English residents in Messina, to be correct; and I attribute it to the physical energy of the constitution being, to a certain degree, impaired by the great heat of the Mediterranean climate, and the effeminate mode of life which the customs of the country oblige them to lead.

On a morning in June, I rode from the Faro Point (Cape Pylorus) to Messina, about eight miles distant, through the thick haze of a strong Sirocco, which, as my servant informed me, had just begun blowing; I never felt its effects so sensibly as upon this occasion. The thermometer was at 94 in the shade. By the time I had reached the lesser lake, near the village of St. Agatha, two miles from the Faro, every thing about me was damp, and the air had apparently lost much of its animating principle, for I was deprived of my usual activity and strength. My face was covered with a clammy moisture, and, although I rode along very leisurely, a profuse perspiration broke out upon my whole person which afterwards produced a chilling effect. Arrived at the city, I found the window-shutters of the houses nearly closed, and only a few mendicants and labourers in the streets, almost all other persons staying within doors till sunset during a violent Sirocco. The thermometer at Messina was at 100, six degrees higher than at the Faro. This wind having ceased on the afternoon of the next day, the thermometer fell in the course of the evening to 90. The barometer sunk two lines. I had no opportunity of observing the state of the hygrometer, but was told by an intelligent capuchin friar that it always indicates considerable humidity of the atmosphere.*

Various other effects are attributed to this peculiar wind; the farmer, the butler, the cook, and the painter lament its effects. Corn and fruit are injured by it at the time of their beginning to vegetate.† Wine continues turbid if bottled, and meat, if salted during a Sirocco, cannot be preserved, nor will paint at this time dry.

---

* "During the blowing of the Sirocco wind, the thermometer uniformly rises three or four degrees, and the hygrometer marks a very great increase of the humidity of the atmosphere, which daily augments while the wind continues. The barometer, at the same time, sinks progressively, and every substance feels damp to the touch."—*Irvine's Letters on Sicily.*

† The Abbate Balsamo has the following passage:—"They (the Sirocco winds) every year occasion, as in Egypt, very great damage in this country (Sicily) to corn and fruit, particularly the wheats, when they blow strongly at the time of their shooting and beginning to vegetate."—See *Vaughan's Translation of the Abbe's Work.*

At Palermo,* Milazzo, and throughout the northern coast of Sicily, this wind is more complained of than in other parts of the island. At Catania, Syracuse, Messina, and Agrigentum, on the eastern and southern shores, it is felt in the succession which those cities are herein mentioned. At Malta and Naples still less. The Sirocco is more boisterous at Messina than at most places, being conducted thither between the hills and mountains of the straits at Cape Pylorus, it is even stronger, the straits being here much narrower than at Messina, and the mountains of either side equally high.

The Sirocco wind certainly hastens the fatal termination of patients in the last stage of consumption, and of persons in whom the high inflammatory fevers of Sicily are attended with great prostration of strength.

Rheumatism is apt to occur during this wind, and, as the late Dr. Irvine observes, old rheumatic pains at this time in-

* "Towards July and August, the cooling western breeze is sometimes interrupted by a Sirocco; this lasts, generally, for about three days, and, though very unpleasant in all parts of the island, it is really intolerable at Palermo, where the inhabitants are literally in a state of partial suffocation during its continuance."—*Blaquiere's Letters from the Mediterranean.*

"I have not been able (says Brydone) to procure any good account of this very singular object in the climate of Palermo. The causes they assign for it are various, though none of them, I think, altogether satisfactory."

"I have seen an old fellow here who has written upon it. He says it is the same wind that is so dreadful in the sandy deserts of Africa, where it sometimes proves mortal in the space of half an hour. He alleges that it is cooled by its passage over the sea, which entirely disarms it of these tremendous effects before it reaches Sicily. But, if this was true, we should expect to find it most violent on that side of the island that lies nearest to Africa, which is not the case; though, indeed, it is possible, that its heat may be again increased by its passage across the island; for it has ever been found much more violent at Palermo, which is near the most northern point, than any where else in Sicily. Indeed, I begin to be more reconciled to this season, when I consider that this city is almost surrounded by very high mountains, the ravines and vallies betwixt which are entirely parched up and burning hot at this season. These, likewise, contain innumerable springs of warm water, the steams of which must tend greatly to increase the heat, and, perhaps, likewise to soften the air and disarm it of its noxious qualities. It is a practice too, at this season, to burn heath and brush-wood on the mountains, which must still add to the heat of the air."—*Tour through Sicily and Malta.*

crease, crease.* About one o'clock in the morning, (July, 1811,) whilst it was blowing a strong Sirocco at the Faro, my regiment having formed in line upon the beach, and piled their arms, some officers and myself lounged upon the deck of a boat which was lying on the sand a little way above the water's-edge; every thing about us became exceedingly damp. We did not, however, remain on the boat more than ten minutes, but paced the beach till daylight with nothing else (in point of fact) to occupy our attention than the Sicilian mariner's watch-word, the English centinel's challenge, and the distant rumble of the enemy's drum. The following evening, the wind still continuing, I had a smart attack of rheumatism of the left shoulder, and three privates were brought to me with the same affection, two of which were chronic cases, attended with considerable augmentation of pain Several instances of these kinds afterwards occurred to me, both at the Faro and in the general hospital of Messina.

I have thought it necessary to make, in the form of notes, some copious extracts from the principal authors on Sicily, as well to confirm what I have stated in respect to the Sirocco wind, as to furnish your readers with almost all that is known concerning it; and, although they may be found to differ in some particulars, yet they all agree (as far as they go) in attributing to it those properties which I have enumerated.

Without indulging myself in abstract speculations on any of the facts contained in this paper, I shall now proceed to offer one or two observations on the climate of Sicily, and on consumption in that kingdom.

---

* "Old rheumatic pains are more severely felt during a Sirocco, which thus agrees in character with its kindred east wind in England. I do not know if it will be accepted as any solution of the cause of this to observe, that the health of our skin, and the right action of the subjacent parts, may not improbably be supposed to be connected, by sympathy or otherwise, with the perfect state of the cutaneous transpiration; but the moisture of the air must unavoidably, upon known chemical principles, impede the formation of vapour upon the surface of the body. When the balance of health is pretty fairly equal among the different parts of the system, the only effect of this may be to excite a general feeling of uneasiness, such as is complained of by so many. But a tender part may be affected more sensibly, and the fluids, refused their usual passage, be forced painfully to distend the yet yielding and flaccid vessels of an organ imperfectly restored to health."—*Dr. Irvine's Letter on Sicily*, vide note at p. 249.

Poets

Poets have ever given to Sicily a climate, which in reality does not belong to her. Tourists have often misrepresented it, either because in their rapid flight through the island they have trusted to the accounts of others, or because they cannot divest themselves of that poetical bias in favour of these classic shores, which, in their youth, they had imbibed from the ancient bards, and which has rendered them blind to every thing but its beauties. Indeed, it was natural to associate a lovely—a perfect clime with Sicilian scenery! The winter of this beautiful isle has been compared (and by the Abbate Balsamo too, the Professor of Agriculture in the University of Palermo,) to a *perpetual spring*. The summer and autumn have been called delightfully *temperate*. In fine, nothing but moonlight nights, without a cloud to deform the face of the starry heavens, cerulean skies, gentle zephyrs, &c. will, in respect to the Sicilian climate, find their way into the writings of poets, of sentimental tourists, and of prejudiced natives. "This climate (says some author) is by no means what we expected to find it, and the serene sky of Italy, so much boasted of by our travelled gentlemen, does not deserve the great eulogiums bestowed upon it."

It is true, the Sicilians cannot complain of the long protracted and severe winter of England, the dense fogs and gloomy weather of our November, nor of those endless clouds from the great western ocean which darken our atmosphere, depress our spirits, and descend upon our isle in drizzly rains; but, with the exception of the spring, which is ever mild and gay and healthful, the climate of Sicily is subject to as frequent variations of temperature as that of England. I have often experienced the four seasons in Sicily in twenty-four hours. The summer is here excessively hot, and almost without a shower of rain to cool the parched ground, or to enliven vegetation. The charming verdure of the fields is at this time lost, and all nature languishes; indeed, this season of the year is so hot that the Messinese are wont to say, "none but Englishmen and dogs expose themselves to the mid-day sun in summer;" the inhabitants retiring from all business between the hours of twelve and two, and, half-closing the window-shutters of their houses, yield themselves up to the luxurious siesta.* The rainy season, with its many, short, and violent showers, is confined to the autumnal months; and both summer and autumn are occasionally visited by the Sirocco. I resided at Messina during the winters of 1810 and 11; and, although my com-

* The mid-day slumber of the Italians and Sicilians.

panion panion and myself built a fire-place* and chimney, and kept up a good fire from the middle of October to the latter end of December, we found ourselves so chilly, even in the parlour, that we were often obliged to huddle on our great coats. Irvine, speaking of the winter in Sicily, says, "The vicissitudes of heat are very great, and I have often seen an alteration of 20° in the temperature in twenty-four hours." Hence, persons in this island, *already debilitated and relaxed by the extreme heat of the summer, and a part of the autumn*, are, when exposed to the rainy, cold, and changeable weather of winter, without well heated stoves, or good fires, or carpets on their *stone* floors, or well fitted window frames, and casements, and doors, as much affected by the cold of the winter in Sicily, (at least for two months) as we are during that period of the same season in England.

Now, when we reflect on the sudden and great changes of weather to which the Sicilians are exposed, we cannot be surprized that phthisis should be found in their bills of mortality; still, as far as my observation went, that lamentable disease was by no means so common in that island as in England. The symptoms of phthisis are oftener mitigated, and their deadly course oftener arrested, than in our own country, and this I think principally arises from the shortness of their winters and their abstemious mode of living.

In consequence of the menacing attitude which Murat, with a large army, had assumed on the opposite shore of Lower Calabria in the spring of 1811, our troops took up positions on the borders and heights of the straits of Messina, and, remaining in them till the following October, engaged by night and day in throwing up batteries and breast-works, or resting upon their arms, in the constant expectation of being invaded, were exposed to the vicissitudes of heat, and subjected to much corporeal fatigue and mental anxiety. Under these circumstances it was that several cases of phthisis occurred amongst our men, towards the November and December of the same year, and of these, a few recovered that would, in my apprehension, have died in England.

The Sicilians desert the consumptive patient, under the idea that phthisis is contagious; and, when he dies, they burn his bed and body-clothes, and ventilate and fumigate the apartments in which he lay.

*Frome; August* 31, 1815.

---

* The inhabitants merely sit in the winter by little charcoal fires, made in chafing-dishes, which were more apt, in our English constitution, to produce nausea and head-ache than warmth.

